# Clinical Features of Malignant Ovarian Germ Cell Tumors According to Demographic and Pathologic Characteristics

**DOI:** 10.1002/hsr2.72334

**Published:** 2026-05-03

**Authors:** Jila Agah, Elham Navipour, Setareh Akhavan, Somaye Norouzi

**Affiliations:** ^1^ Non‐Communicable Diseases Research Center Sabzevar University of Medical Sciences Sabzevar Iran; ^2^ Iranian Research Center on Healthy Aging Sabzevar University of Medical Sciences Sabzevar Iran; ^3^ Department of Obstetrics and Gynecology, School of Medicine, Vali Asr Hospital Tehran University of Medical Sciences Tehran Iran

**Keywords:** clinical features, demographic characteristics, malignant ovarian germ cell tumors, pathology

## Abstract

**Background and Aims:**

Malignant ovarian germ cell tumors (MOGCTs) are aggressive cancers affecting mainly young women, in whom fertility preservation is important. Diagnosis is often delayed because early symptoms are non‐specific, leading to advanced disease or emergency surgery that may limit optimal staging and fertility‐sparing treatment. As no effective screening exists and early detection improves survival, increased symptom awareness is essential. This study describes the demographic and clinical characteristics of MOGCTs to support earlier diagnosis.

**Methods:**

The present study is a descriptive–analytical cohort study conducted at Vali‐ASR Hospital in Tehran, Iran, from 2001 to 2018. Patients with malignant ovarian tumors were evaluated with respect to demographic characteristics, obstetric and medical history, pathological findings, and clinical signs and symptoms before and at the time of diagnosis. The duration of symptoms was also recorded. Data was analyzed using SPSS version 24, employing descriptive statistical methods.

**Results:**

The mean age of MOGCT cases (128 ones) was 23.88 ± 7.85 years. We found abdominal distension (45%) followed by acute pain (40.95%), chronic pain (23.95%), menstrual irregularity (14.7%), sense of abdominal firmness and mass (7.72%), nausea (5.4%), fever (5.4%), lack of apatite (4.63%), virilization (3.1%), and depletion of weight (3.1%). Abdominal distension and pain, acute or chronic, and menstrual disorders were the most common symptoms in all patients, but their incidence declined along with aging. The percentage of abdominal distention, ascites, and menstrual disorders in parous women was less than that of nullipara.

**Conclusion:**

MOGCTs present a significant diagnostic challenge due to their nonspecific and often misinterpreted symptoms, which vary notably by histologic subtype. Our findings emphasize that a high index of suspicion, coupled with an understanding of this symptom variability, is crucial for the timely diagnosis and improved management of these aggressive tumors, particularly in young women.

## 1 Introduction

Ovarian cancer is the second most prevalent gynecologic malignancy in both the United States and developing countries [1, 2], and notably, it remains the most lethal gynecologic cancer [3]. A critical challenge is the lack of effective screening tools for the general population, which contributes to its high lethality and significant economic burden. The cost of treatment per patient is the highest among all cancer types; for example, the average initial cost in the first year can amount to around $80,000, rising to approximately $ 100,000 in the final year [4]. Consequently, developing cost‐effective strategies for early detection and prevention has been a major focus of research over the last decade [4]. In 2018, an estimated 22,240 new cases were diagnosed, and 14,070 deaths occurred due to the disease in the United States [5].

Currently, CA125 and HE4 are the only approved biomarkers, yet they are insufficient for early detection. To mitigate the limitations of single serum biomarkers, especially in the pre‐surgical evaluation of adnexal masses, multivariate index (MVI) assays have been developed. Among these, the risk of malignancy algorithm (ROMA) integrates menopausal status with CA125 and HE4 concentrations. Furthermore, miRNAs show remarkable potential for ovarian cancer prediction, though their characterization as biomarkers requires further standardization of sample processing and refinement of detection platforms [6].

Ovarian neoplasms are classified based on histologic origin into four major categories: epithelial tumors, germ cell tumors, sex cord‐stromal tumors, and ovarian carcinosarcomas (mixed malignant Müllerian tumors) [7, 8].

Carcinosarcomas are rare, biphasic, and challenging tumors accounting for only 1%–4% of ovarian cancers. They follow a distinct, aggressive natural history with a dismal prognosis, and most patients relapse within a year of initial treatment [8]. Germ cell tumors, the focus of this study, account for 15%–20% of all ovarian neoplasms and predominantly occur during adolescence and early adulthood [9]. Malignant ovarian germ cell tumors (MOGCTs) are most prevalent in Central America and Eastern Asia [10].

The prognosis of ovarian cancer strongly depends on the stage at initial diagnosis, yet over 70% of patients present with advanced‐stage disease (beyond the pelvis) at detection [4, 11, 12]. For epithelial ovarian cancer, *BRCA1/2* germline mutations represent the most significant known genetic risk factors, occurring in approximately 6%–15% of affected women. *BRCA1/2* status provides valuable prognostic information, as carriers tend to exhibit greater sensitivity to platinum‐based chemotherapy, leading to improved survival despite often being diagnosed at an advanced stage and higher grade [13].

Although ovarian cancer is often described as a “silent killer,” most patients experience symptoms before diagnosis, though these are frequently non‐gynecologic [14]. Patients frequently report nonspecific abdominal, pelvic, gastrointestinal, and urinary symptoms over a prolonged period [15], which are often ignored by patients or misdiagnosed by physicians [16]. Notably, symptom type does not significantly differ between early and late‐stage disease. The 5‐year survival for early‐stage disease is 70%–90% compared with 20%–30% for advanced‐stage disease [17]. Despite attempts to develop multimodal screening methods to reduce mortality, no approach has been approved [18]. Therefore, recognizing early symptoms and detecting early‐stage disease remain crucial for improving patient outcomes [19].

This is particularly critical for MOGCTs. They often lack specific symptoms and may first present as an acute abdomen [20, 21], warranting emergency laparotomy. During such emergencies, complete staging and optimal cytoreductive surgery may be missed, compromising overall and progression‐free survival. Furthermore, for these young patients, fertility‐preserving surgery is a key consideration. In this context, second‐look surgery should be considered for patients with incompletely resected tumors containing teratomatous elements, typically reserved for residual immature teratoma after chemotherapy or growing teratoma syndrome [22]. Although MOGCTs are uncommon in older adults, postmenopausal women with an ovarian mass and an elevated serum AFP level should also be suspected of a germ cell tumor [23]. Early‐stage diagnosis makes fertility‐sparing and optimal surgical management more feasible [22, 24, 25]. Notably, many patients report uncomfortable feelings for days or weeks prior to acute presentation, which are unfortunately often ignored [26]. Given the limited literature and the importance of symptom awareness for the early detection of MOGCTs, this study aimed to characterize their clinical features based on demographic and pathological data.

## 2 Methods and Materials

### 2.1 Study Design, Setting, and Sample Size

**Figure 1:**
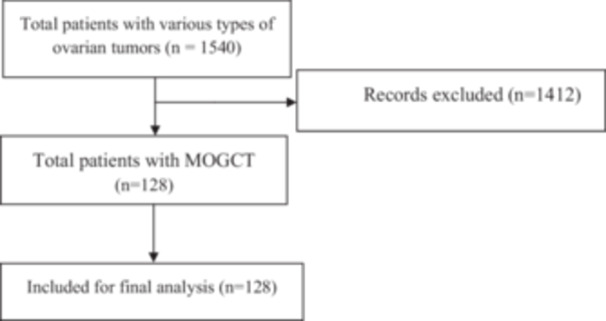
STROBE flow diagram.

This cross‐sectional study aimed to identify clinical features of malignant ovarian germ cell tumors based on demographic and pathological data from 2001 to 2018. This study was conducted in Vali‐ASR hospital, Tehran, Iran. It is the first and main referral center of gynecologic oncology in Iran. After obtaining approval from the medical school of Tehran University, all the patients with malignant ovarian tumors were evaluated and were detected and investigated. Of the 1540 patients having various types of ovarian tumors, 128 cases of MOGCTs were detected and enrolled in this study (Figure 1).

### 2.2 Study Instruments

All the information about demographic features, obstetrical and medical history, pathologic reports, symptoms and signs, and duration of symptoms was obtained through study of hospital cases and interviews with the affected women and their close relatives. It was completed using a questionnaire containing demographic information such as age, education, body mass index, puberty, marital, and parity status. Also, all symptoms and signs, including abdominal distention, pain, acute or chronic, menstrual irregularity, sense of mass, nausea, urinary symptoms, fever, lack of appetite, virilization, and weight depletion were recorded. Also, the patients whose tumors had been detected incidentally while they were asymptomatic were detected. Meanwhile, the patients were divided according to age groups (age ≤ 19, 20 ≤age≤ 29, age ≥ 30), parity status, and pathologic subtypes.

### 2.3 Data Analysis

Data (qualitative and quantitative) were coded and analyzed in SPSS 24. Symptom prevalence was assessed across all MOGCT cases and stratified by pathological subtype, age group, and parity status. Descriptive statistics for demographic variables are reported as frequencies (*n*) and percentages (%). Statistical significance was defined a priori as a two‐sided *p* value < 0.05.

## 3 Results

**Figure 2:**
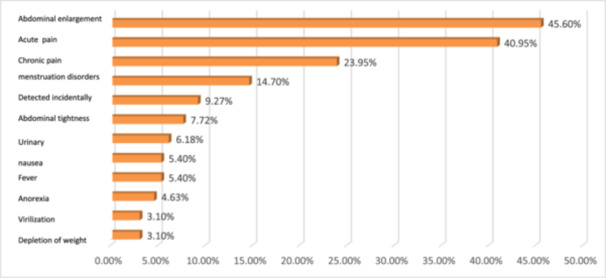
Frequency symptoms and signs in the whole ovarian germ cell.

The mean age of MOGCT cases (128 cases) was 23.88 years. Fifty‐two point three percent were married, 68.8% had no parity, and 7.8% were pregnant when the tumor was detected (Table 1). In MOGCT cases, abdominal distension and weight depletion were respectively the most and the least common symptoms. 9.27% were asymptomatic and detected incidentally. About 41% referred first by symptoms of acute abdomen (Figure 2). The percentage of teenagers was 33.6%, the age group of 20–29 years at 44%, and ≥ 30 years at 22.4%. Although abdominal distension and pain, acute or chronic, and menstrual disorders were the most common symptoms in all age groups, their incidence declined along with aging (Table 2). Anyway, abdominal distension and pain were the most common symptoms in both groups. Asymptomatic cases were more in parous women and in patients whose tumors were distinguished during pregnancy (Table 3). Incidence of subtypes of MOGCTs was, respectively, dysgerminoma (35.2%), immature teratomas (26.4%), mixed (15.2%), yolk sac (13.6%), Scc.teratoma [4], undifferentiated (2.4), embryonal (1.6), choriocarcinoma (0.8), carcinoid (0.8). Abdominal distension and pain were the most common symptoms in all subtypes. Dysgerminoma and immature teratoma were the most common types, which were detected incidentally. Menstrual disorders and sense of mass were more in dysgerminoma and mixed (Table 4). Eighty‐five percent of the patients stated that they had symptoms for several days or weeks and even months before surgery, but had been ignored by themselves or managed by their physicians. Dysgerminoma and immature teratoma for 3 days–11 months, mixed tumors for 15 days –11 months, yolk sac for 7 days–2 months, and scc.teratoma for 4 months were reported by the patients. Seventeen percent had no menstruation, which was prepubertal or had dysgenetic gonads. Five cases showed xy in the karyotype.

**Table 1:** Baseline characteristics.

Symptom	*n* (%)	
Age	Min (year)	11
	Mean (mean ± SD)	23.99 ± 7.88
	Max (year)	50
Education	Student (school and university)	72.4%
	Others	27.6%
Place of living	Urban	80%
	Rural	20%
Socio‐economic status	Well‐off	15.4%
	Average	76.9%
	Poor	7.7%
Body mass index	Thin	25%
	Normal	53.6%
	Fat	21.4%
Marital status	Single	47.7%
	Married	52.3%
Parity	No parity	68.8%
	Parous	31.2%
Pregnant	%7.8	
Menstruation	Yes	106 (82.8%)
	No	22 (17.2%)
Menstruation disorders	Yes	43 (40.57%)
	No	63 (59.43%)

**Table 2:** Frequency of symptoms in age groups.

Symptoms	Age		
	≤ 19: (*N* (%))	20–29: (*N* (%))	30 ≤: (*N* (%))
Abdominal enlargement	27 (64.3%)	21 (38.2%)	8 (28.6%)
Acute pain	16 (38.1%)	24 (43.6%)	10 (35.7%)
Menstrual disorders	7 (16.7%)	9 (16.4%)	2 (7.1%)
Chronic pain	7 (16.7%)	13 (23.6%)	9 (32.1%)
Ascites	7 (16.7%)	4 (7.3%)	1 (3.6%)
Abdominal tightness	6 (14.3%)	4 (7.3%)	0 (0%)
Nausea	5 (11.9%)	1 (1.8%)	1 (3.6%)
Urinary	3 (7.1%)	2 (3.6%)	3 (10.7%)
Torsion	2 (4.8%)	4 (7.3%)	2 (7.1%)
Fever	2 (4.8%)	4 (7.3%)	1 (3.6%)
Anorexia	1 (2.4%)	4 (7.3%)	1 (3.6%)
Depletion of weight	1 (2.4%)	2 (3.6%)	1 (3.6%)
Hemoperitoneum	0 (0%)	2 (3.6%)	0 (0%)
Virilization	0 (0%)	3 (5.5%)	1 (3.6%)
Detected incidentally	0 (0%)	3 (5.5%)	5 (17.9%)

**Table 3:** Frequency of symptoms according to parity.

Symptoms	Nulliparous women (*N* (%))	Parous women (*N* (%))	During pregnancy (*N* (%))
Abdominal enlargement	43 (48.9%)	14 (35%)	2 (20%)
Acute pain	34 (38.6%)	16 (40%)	3 (30%)
Chronic pain	22 (25%)	8 (20%)	1 (10%)
Menstrual disorders	17 (19.3%)	2 (5%)	—
Ascites	10 (11.4%)	2 (5%)	—
Abdominal tightness	9 (10.2%)	1 (2.5%)	—
Urinary	7 (8%)	1 (2.5%)	—
Fever	6 (6.8%)	1 (2.5%)	—
Nausea	6 (6.8%)	1 (2.5%)	—
Torsion	5 (5.7%)	3 (7.5%)	—
Anorexia	4 (4.5%)	2 (5%)	—
Depletion of weight	4 (4.5%)	—	—
Virilization	2 (2.3%)	2 (5%)	—
Detected incidentally	1 (1.1%)	7 (17.5%)	4 (40%)
Hemoperitoneum	1 (1.1%)	1 (2.5%)	—

**Table 4:** Symptoms according to histology.

Symptoms	Undeferentiated (*N* (%))	Mixed (*N* (%))	Scc. teratoma (*N* (%))	Carcinoid (*N* (%))	Choriocarcinoma (*N* (%))	Embryonal (*N* (%))	Yolk sac (*N* (%))	Immature teratoma (*N* (%))	Dysgerminoma (*N* (%))
Abdominal enlargement	—	7 (36.8%)	3 (60%)	—	—	2 (100%)	8 (47.1%)	14 (42.4%)	22 (50%)
Acute pain	3 (100%)	8 (42.1%)	2 (40%)	—	1 (100%)	1 (50%)	8 (47.1%)	15 (45.5%)	11 (25%)
Chronic pain	—	4 (21.1%)	2 (40%)	—	1 (100%)	—	4 (23.5%)	9 (27.3%)	9 (20.5%)
Menstrual disorders	—	4 (21.1%)	—	—	—	—	1 (5.9%)	5 (15.2%)	9 (20.5%)
Abdominal tightness	—	3 (15.8%)	—	—	—	—	1 (5.9%)	1 (3%)	5 (11.4%)
Ascites	—	2 (10.5%)	—	—	—	—	4 (23.5%)	1 (3%)	5 (11.4%)
Urinary	—	—	1 (20%)	—	—	—	2 (11.8%)	1 (3%)	4 (9.1%)
Detected incidentally	—	1 (5.3%)	—	—	—	—	—	4 (12.1%)	3 (6.8%)
Torsion	—	—	—	—	—	—	2 (11.8%)	4 (12.1%)	2 (4.5%)
Nausea	—	2 (10.5%)	—	—	—	—	1 (5.9%)	2 (6.1%)	2 (4.5%)
Virilization	—	1 (5.3%)	—	—	—	—	1 (5.9%)	—	2 (4.5%)
Depletion of weight	—	1 (5.3%)	—	—	—	—	1 (5.9%)	—	2 (4.5%)
Anorexia	—	1 (5.3%)	—	—	—	—	1 (5.9%)	3 (9.1%)	1 (2.3%)
Fever	—	1 (14.3%)	—	—	—	—	3 (17.6%)	2 (6.1%)	1 (2.3%)
Hemoperitoneum	—	—	—	—	—	—	1 (5.9%)	—	1 (2.3%)

More than 90% showed abdominal distension and/or pain. About 41% referred to acute abdomen, although only 15% had sudden acute pain, and 26% stated mild nonspecific symptoms in previous days. Hormonal disorder is seen as menstrual irregularity (14.7%) and virilization (3.10%). Acute abdomen was more than distention in women older than 30 years, but the size of mass was smaller after 30 years compared to younger women. The percentage of abdominal distention, ascites, and menstrual disorders in parous women was less than that of nulliparae. The three undifferentiated cases referred only to acute abdomen. Torsion of mass in immature and yolk sac was more, and fever was higher in immature and mixed tumors.

## 4 Discussion

As MOGCTs represent a small subset of ovarian germ cell neoplasms, interest in their clinical and histologic characteristics has declined over the past two decades. However, they still comprise a notable proportion of ovarian tumors in regions such as Asia and Africa [27, 28, 29, 30], with our study identifying a prevalence of 8.3%. Affected individuals were typically young women in their second or third decade of life, commonly presenting with progressive abdominal distension, often accompanied by pain.

As the ovary enlarges in a woman with ovarian neoplasm, there may be progressive compression of the surrounding pelvic structures, which leads to vague abdominal discomfort, pelvic pressure, and urinary or gastrointestinal symptoms. Usually, abdominal enlargement becomes apparent whenever the neoplasm reaches up to 10–15 cm. Although even in this phase, the symptoms may be attributed to gas or indigestion by either the patient and/or the physician in primary care services [31]. Beforehand, it was believed that ovarian cancer is an asymptomatic disease [32], because frequently it is recognized at advanced stages [33].

A review study showed that while approximately 95% of all patients experience nonspecific symptoms, such as abdominal pain, bloating, and urinary urgency, about 80% are diagnosed with advanced‐stage disease (stage III–IV), which includes extra pelvic disease, ascites, and abdominal masses prior to diagnosis [34]. However, the most common symptoms were abdominal or gastrointestinal, whereas gynecologic symptoms were the least common [20]. In our study, 90.6% of cases had symptoms prior to diagnosis. Lack of symptoms was more prevalent in older and/or parous women compared to other subjects. Of the 12 cases (9.4%) who were asymptomatic, 30% (4/12) were pregnant, whose tumors had been detected incidentally during routine ultrasonography. We found abdominal distension (45%) as the most common symptom followed by acute pain (40.95%), chronic pain (23.95%), menstrual irregularity (14.7%), sense of abdominal firmness and mass (7.72%), nausea (5.4%), fever (5.4%), lack of appetite (4.63%), virilization (3.1%), and depletion of weight (3.1%). Evaluations according to age groups showed that in younger patients, particularly teenagers, abdominal distention is the most prominent sign. It correlates with the location of the ovary in the abdomen in children and very young girls and the limited outlines of the bony pelvis, which permits only upward expansion of the tumor. This may explain why the pain is classically periumbilical in these patients [35, 36]. Also, we found that the distention or firmness of the abdomen and sense of mass in nulliparous women were more than in multipara, which may be due to abdominal enlargement and muscle weakness, which is usually seen in parous women that can obscure pathologic conditions [26].

A report from India showed pain as the most common symptom (77%), followed by distension of abdomen 45.5%, sense of mass (7.8%), menstrual irregularities (16.9%), urinary complaints (5.2%), and bowel complaints (3.9%) [37]. Based on some other reports, pain is the most common symptom in MOGCTs (55%–80%), followed by abdominal mass and abdominal pain associated with a palpable mass have been reported in 85% of cases [38, 39]. Pain may be chronic and vague for a duration of days or even weeks, or acute with an urgent presentation. This acute event frequently happens due to cystic rupture or torsion and infection, and may be accompanied by ascites or hemoperitoneum [39]. Some authors report that pain is chronic in nature, but in up to 30% of cases, it may be aggravated as an acute abdomen, which mandates urgent exploration surgery for presumed acute appendicitis [38]. To verify, we faced a high presentation of acute abdomen (41%), which was clearly more than previous reports. Following an exact interview with our patients, we found that only 15% of them had suddenly complained of merely acute pain, whereas the others (26%) stated some nonspecific symptoms in previous days, which unfortunately had been ignored. Notably, we had three undifferentiated germ cell tumors, all of them referred to with acute abdomen. It may be due to the highly rapid progression of these tumors.

Fever is another sign that has been reported in 10%–25% of cases [39], although it was observed in only 5.4% of our study population. Menstrual disorders, such as vaginal bleeding and irregularity, were noted in 9.27% of our cases, which aligns with previous reports citing a prevalence of 10% [29] but contrasts with findings from other studies reporting a lower incidence of approximately 5% [40, 41, 42, 43]. These symptoms were more common among nulliparous and younger women.

Histological subdivision shows that abdominal distention and pain are the most common symptoms in all subtypes, with slightly different values. In dysgerminoma, which is the most prevalent type of MOGCTs, 50% had distension and 25% had acute pain, which disagrees with other reports that tell acute abdomen is uncommon [44], and one‐fifth of patients had menstrual disorders, which can be due to HCG secretion [45]. This type of hormone can induce hirsutism, which we observe in two people.

The most common symptom in immature teratoma was an acute abdomen, and 12% showed torsion of the tumor. Sometimes, it is associated with para‐neoplastic syndrome, such as limbic encephalitis [46], which we did not observe in our patients. Nine percent of our cases complained of a lack of appetite, which was more than other subtypes. Of note, asymptomatic cases were more prevalent in immature teratoma compared to other pathologies.

The patients with yolk sac tumor showed abdominal distention (47%) and acute abdomen in a high percentage at 47%, 11% had torsion of tumor, 23.5% had ascites, and 17.6% had fever, which were apparently more than other subtypes. This tumor can be associated with gonadal dysgenesis and virilizing symptoms [47], which we observed in one person.

The most common clinical presentation of mixed tumors includes an abdominal mass with or without abdominal pain or fever [48]. We found acute abdomen as the most common symptom (42.1%), followed by abdominal distension (36%) and sense of abdominal firmness and mass (15.8%), nausea (10.5%), and fever (14.5%) were prominent in comparison to other types. Absolutely, the histology existing in the tumor influences present symptoms. According to literature, most mixed germ cell tumors consist of a combination of dysgerminoma with yolk sac tumor accounting for one‐third of the cases [48]. The structure of yolk sac tumors resembles the primitive yolk sac and exhibits a variety of histological patterns, including microcystic/reticular, endodermal sinus (festoon), solid, alveolar‐glandular, parietal, papillary, polyvesicular vitelline, hepatoid, and myxomatous [49]. We found the combination of yolk sac and immature teratoma as the most common combination (15.8%). Sporadically, dysgerminoma and yolk sac were observed in 55% of our cases. Some components of mixed tumors may secrete estrogen and can present precocious puberty or irregular vaginal bleeding [4] or secrete androgens, causing virilizing symptoms [48]. Twenty‐one percent of our cases showed vaginal bleeding, and 5% showed hirsutism. We found five cases of 46xy in our patients, of which three were mixed, and two had dysgerminoma tumors and had no menstruation. Gonadal dysgenesis is reported to be 1 per 80,000 births, and primary amenorrhea is the most common symptom [50, 51]. The risk of malignancy in dysgenetic gonads is increased up to 30%–50% [52, 53].

Malignant transformation of teratoma is an uncommon complication and is observed in 1%–2% of cases. The most common histology is squamous cell carcinoma (scc). Most of them are premenopausal women and are asymptomatic or present with nonspecific complaints (teratoma). We had five cases of scc teratoma who had manifestations of abdominal distension (60%), acute abdomen (40%), chronic pain (40%), and urinary symptoms (20%).

## 5 Conclusion

MOGCTs, though rare globally, remain clinically significant in regions like Asia and Africa. Our study highlights their prevalence at 8.3%, predominantly affecting young women who often present with abdominal distension, pain, and nonspecific gastrointestinal symptoms. These signs are frequently misinterpreted, delaying diagnosis. Acute abdomen was notably common, especially in undifferentiated and mixed tumors, suggesting aggressive behavior. Symptom patterns varied by histologic subtype, with dysgerminomas linked to menstrual irregularities and hormonal effects, while yolk sac tumors showed higher rates of ascites and fever. Mixed tumors demonstrated diverse presentations due to their composite nature. Notably, asymptomatic cases were more frequent in immature teratomas and among pregnant women. Our findings underscore the importance of early recognition, thorough clinical evaluation, and awareness of symptom variability to improve diagnostic accuracy and outcomes in MOGCTs, especially in younger and nulliparous women.

## Author Contributions

**Jila Agah:** conceptualization, investigation, methodology, data curation, supervision, formal analysis, validation, writing – original draft, writing – review and editing, project administration. **Elham Navipour:** software, data curation, formal analysis, writing – review and editing, writing – original draft. **Setareh Akhavan:** conceptualization, methodology, investigation, writing – original draft, writing – review and editing. **Somaye Norouzi:** methodology, software, formal analysis, writing – original draft, writing – review and editing.

## Funding

The authors have nothing to report.

## Ethics Statement

The study was conducted in accordance with the Helsinki Declaration and the Committee on Publication Ethics (COPE) guidelines. Ethical approval was obtained from the Tehran University of Medical Sciences Ethics Committee (IR.TUMSJKHC.Rec.1396.4819).

## Consent

Written and verbally informed consent was obtained from all participating patients, who were free to withdraw at any time. Participation was voluntary, and all questionnaires were completed anonymously. Data confidentiality was strictly maintained.

## Conflicts of Interest

The authors declare no conflicts of interest.

## Transparency Statement

The corresponding author, Somaye Norouzi, affirms that this manuscript is an honest, accurate, and transparent account of the study being reported; that no important aspects of the study have been omitted; and that any discrepancies from the study as planned (and, if relevant, registered) have been explained.

## Data Availability

The data sets used and/or analyzed in the current study are available from the corresponding author upon reasonable request.
